# High Seroprevalence of Antibodies to Avian Influenza Viruses among Wild Waterfowl in Alaska: Implications for Surveillance

**DOI:** 10.1371/journal.pone.0058308

**Published:** 2013-03-05

**Authors:** Heather M. Wilson, Jeffery S. Hall, Paul L. Flint, J. Christian Franson, Craig R. Ely, Joel A. Schmutz, Michael D. Samuel

**Affiliations:** 1 United States Fish and Wildlife Service, Migratory Bird Management, Anchorage, Alaska, United States of America; 2 United States Geological Survey, National Wildlife Health Center, Madison, Wisconsin, United States of America; 3 United States Geological Survey, Alaska Science Center, Anchorage, Alaska, United States of America; 4 United States Geological Survey, Wisconsin Cooperative Wildlife Research Unit, University of Wisconsin, Madison, Wisconsin, United States of America; Thomas Jefferson University, United States of America

## Abstract

We examined seroprevalence (presence of detectable antibodies in serum) for avian influenza viruses (AIV) among 4,485 birds, from 11 species of wild waterfowl in Alaska (1998–2010), sampled during breeding/molting periods. Seroprevalence varied among species (highest in eiders (*Somateria* and *Polysticta* species), and emperor geese (*Chen canagica*)), ages (adults higher than juveniles), across geographic locations (highest in the Arctic and Alaska Peninsula) and among years in tundra swans (*Cygnus columbianus*). All seroprevalence rates in excess of 60% were found in marine-dependent species. Seroprevalence was much higher than AIV infection based on rRT-PCR or virus isolation alone. Because pre-existing AIV antibodies can infer some protection against highly pathogenic AIV (HPAI H5N1), our results imply that some wild waterfowl in Alaska could be protected from lethal HPAIV infections. Seroprevalence should be considered in deciphering patterns of exposure, differential infection, and rates of AIV transmission. Our results suggest surveillance programs include species and populations with high AIV seroprevalences, in addition to those with high infection rates. Serologic testing, including examination of serotype-specific antibodies throughout the annual cycle, would help to better assess spatial and temporal patterns of AIV transmission and overall disease dynamics.

## Introduction

The role of wild birds in the spread of highly pathogenic avian influenza virus (HPAIV) H5N1 has been widely debated [1], but waterfowl have consistently represented the primary reservoir of avian influenza viruses (AIV) across the globe [2]. Experimental inoculations with HPAIV H5N1 in waterfowl have shown that the susceptibility, development of clinical signs, and frequency of mortality varies among and within species [3], [4]. These results have been confirmed by relatively low mortality rates observed during some HPAIV H5N1 outbreaks and by isolation of the virus from surviving birds [5]–[7]. Recent research with captive birds demonstrates increased ability to survive HPAIV H5N1 infection in individuals with certain low pathogenic avian influenza virus (LPAIV) antibodies formed via previous exposure [8]–[13]. If the cross-protective properties of these LPAIV antibodies increase a bird's probability of surviving HPAIV H5N1infection, then theoretically, a portion of surviving birds could contribute to the potential spread of the disease. However, some experimental studies have also shown that birds with LPAIV antibodies can exhibit reduced magnitude and duration of shedding when infected with other LPAIV's or HPAIV H5N1 [10], [13], [14], thereby decreasing their likelihood of facilitating further transmission. Understanding the balance of these elements is an area of active research, but the primary concern still stands: if previous immunity (i.e., pre-existing antibodies) results in lower HPAIV H5N1 pathogenicity, and surviving birds can migrate while infectious, they could play an important role in HPAIV H5N1 disease dynamics and infection rates.

Results of recent surveillance in Alaska suggest that infection with AIV is relatively rare and/or difficult to detect. Shedding of LPAIV in wild Alaska birds was found in only 0.06% of individuals sampled between 1998 and 2004 (*n* = 8,254 samples; [15]) and 1.7% during May 2006 and March 2007 (*n* = 16,797; [16]), using real-time reverse transcriptase polymerase chain reaction (rRT-PCR) and virus isolation from cloacal swabs or fecal samples. Yet, given the short duration of AIV shedding in birds, the window of opportunity for detecting infection is limited [17], therefore, shedding rates alone may be a substantial underestimate of the likelihood of AIV infection [18]. In contrast, the occurrence of AIV antibodies in waterfowl, developed as a response to viral infection, may remain detectable in the blood for many months, offering a much longer time period to assess AIV exposure [14], [18], [19].

Our study examined seroprevalence of AIV antibodies across a range of wild waterfowl species and among a variety of locations and years in Alaska. Our objective was to assess previous AIV exposure, and thus, the proportion of birds with some potential to develop inapparent HPAIV infections (i.e., birds that might be able to become infected and shed HPAIV, without debilitating or fatal illness). Additionally, we sought to examine broad patterns of variation in AIV seroprevalence among species, sexes, ages, geographic locations, and years. Our goal was to better understand how these variables might influence the spatial and temporal dynamics of AIV, in order to help guide future AIV surveillance.

## Materials and Methods

### Ethics Statement

All animal sampling (i.e., swabs and serum collection) was accomplished according to protocols approved by the Institutional Animal Care and Use Committee (IACUC) of the U.S. Fish and Wildlife Service Region 7, and the U.S. Geological Survey (USGS). All birds were captured, sampled, and subsequently released in the wild under U.S. Department of the Interior, USGS Federal Bird Banding Permits #20022 and 22453.

### Serum collection and processing

**Figure 1:**
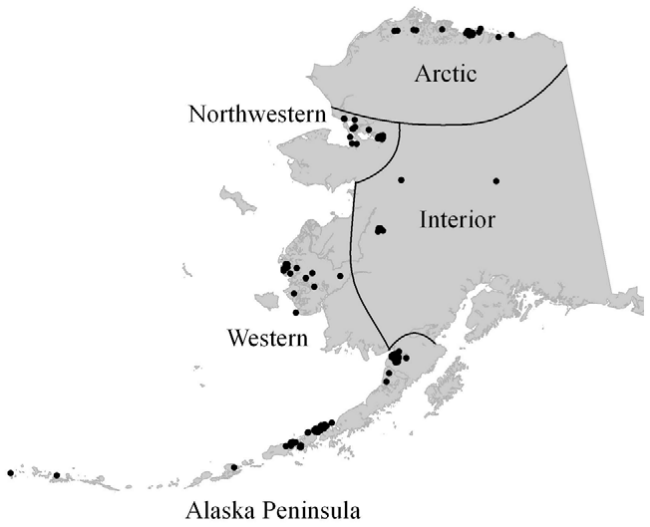
Geographic locations of avian influenza seroprevalence sampling sites in Alaska. Black dots indicate specific sampling sites. Labels delineate generalized regions for analysis.

We collected serum samples from 11 species of wild waterfowl, including: tundra swans (*Cygnus columbianus*), cackling geese (*Branta hutchinsii*), Pacific black brant (*B. bernicla nigricans*), greater white-fronted geese (*Anser albifrons*), emperor geese (*Chen canagica*), northern pintails (*Anas acuta*), Pacific common eiders (*Somateria mollissima v-nigrum*), spectacled eiders (*S. fischeri*), Steller's eiders (*Polysticta stelleri*), long-tailed ducks (*Clangula hyemalis*), and black scoters (*Melanitta nigra*), across five geographic regions in Alaska: the Arctic (Arctic Coastal Plain), Northwestern (NW; Kotzebue area), Western (W; Yukon-Kuskokwim Delta), Interior (IN), and Alaska Peninsula (AP; including the Aleutian Islands), between 1998 and 2010 (Fig. 1).

Individual birds were primarily captured on or near nests (June-July), from flightless molting flocks (July-August), or on autumn staging areas (September). From each bird, we collected up to 6 ml of blood by jugular or brachial venipuncture. Immediately following collection of samples, we transferred blood to sterile Vacutainers® equipped with serum separators, allowed the blood to clot for a minimum of 2 h, and then centrifuged blood at 3000 rpm for up to 10 min. Serum was removed from clotted samples and transferred to cryovials, frozen in liquid nitrogen vapor shippers (−150°C), and maintained frozen at −80°C in a laboratory until testing. We used a commercial blocking enzyme-linked immunosorbent assay (bELISA, FlockChek® AI MultiS-Screen Antibody Test Kit, IDEXX Laboratories, Westbrook, Maine, USA), to detect AIV antibodies [20], [21] according to the manufacturer's directions. Validation data provided by the manufacturer reported this test procedure having a sensitivity of 95.4% and specificity of 99.7%, across a range of domestic waterfowl and poultry. From 2006–2010, many of the species included in our study were also sampled and tested for virus shedding (using rRT-PCR and virus isolation) in conjunction with a larger AIV surveillance program in Alaska [16], [22].

### Statistical Analyses

We used logistic regression within an information theoretic framework to examine variation in AIV seroprevalence among species, sexes, ages, years, and geographic locations. Because our data were collected opportunistically across a variety of years and locations, we took a tiered approach, conducting analyses at increasingly finer scales, as the data allowed. First, we examined effects of species and sex using data from adults. Second, we analyzed the effect of age, only for those species in which adults and sub-adults were sampled (tundra swans, greater-white fronted geese, Pacific black brant, and northern pintails). We created two age classes; “sub-adult” which represented hatch year (HY) birds (∼2–6 mo. old) for northern pintails and second year (SY) birds (∼13–17 mo. old) for all other species, and “adults” which represented after hatch year (AHY) birds for northern pintails and/or after second year (ASY) birds for other species. Third, we examined annual and geographic variation in seroprevalence rates for 3 species (tundra swans, greater white-fronted geese, and northern pintails) for which we had sufficient samples across geographic regions within and/or across years. Based on results from the larger dataset, we included age and sex in all our models of annual and geographic variation in seroprevalence. We examined one and two-way effects for all the covariates, and used Akaike's Information Criterion (AIC), ΔAIC, and model weights (*w_i_*; the likelihood that each model is the best within the candidate suite based on a balance between fit and parsimony) to evaluate strength of evidence for competing models [23]. Models were ranked and top models selected according to lowest AIC value and highest *w_i_* relative to others in the model suite. To better understand relationships between explanatory variables and seroprevalence rates, we report odds ratios and confidence intervals from our top models and give actual percentages of AIV antibody positives within groups of interest.

## Results

**Figure 2:**
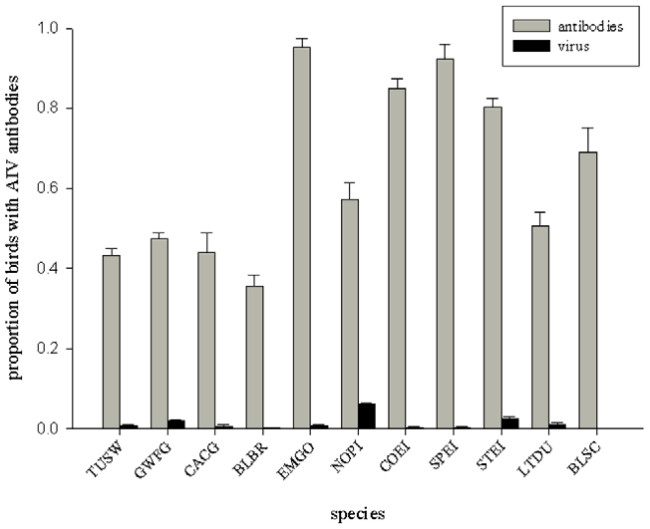
Overall prevalence rates (± s.e.) of avian influenza virus antibodies (gray bar) and avian influenza virus detection (black bar; based on pooled cloacal and oral-pharyngeal swabs; Ip et al. 2008, USGS/USFWS 2009, 2010) for adult waterfowl species in Alaska. Species abbreviations: TUSW  =  Tundra swan (*Cygnus columbianus*), CACG  =  cackling goose (*Branta hutchinsii*), BLBR  =  Pacific black brant (*B. bernicla nigricans*), GWFG  =  greater white-fronted goose (*Anser albifrons*), EMGO  =  emperor goose (*A. canagica*), NOPI  =  northern pintail (*Anas acuta*), COEI  =  Pacific common eider (*Somateria mollissima v-nigrum*), SPEI  =  spectacled eider (*S. fischeri*), STEI  =  Steller's eider (*Polysticta stelleri*), LTDU  =  long-tailed duck (*Clangula hyemalis*), and BLSC  =  black scoter (*Melanitta nigra*).

We tested a total of 4,459 serum samples, 1,968 of which were positive for AIV antibodies, resulting in an overall seroprevalence of 44%. Independent, large-scale AIV monitoring in Alaska revealed few infectious birds (those shedding AIV) among the species we sampled for antibodies (range: 0–7%; Fig. 2; [16], [22]). Adult emperor geese and all three eider species had the highest seroprevalence rates (80–95%; Fig. 2) in our study, while black brant had the lowest (36%; Fig. 2). Among duck species, long-tailed ducks had the lowest seroprevalence (51%), followed by northern pintails (57%), and black scoters (69%), while the three species of eiders (the most marine-dependent of duck species sampled) averaged 86% (Fig. 2). With the exception of emperor geese, other swans and geese had markedly lower seroprevalence rates than ducks (43% vs. 72%; Fig. 2).

**Figure 3:**
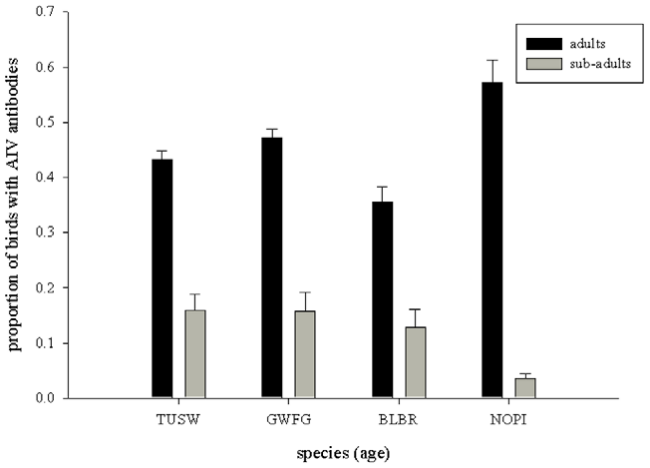
Age differences in seroprevalence rates (± s.e.) of avian influenza virus (AIV) antibodies between adult and sub-adult tundra swans (TUSW), greater white-fronted geese (GWFG), black brant (BLBR), and northern pintails (NOPI) in Alaska. Overall, adults had.7.7 (95% CI: 5.7–10) times greater probability of having AI antibodies than did sub-adults.

**Figure 4:**
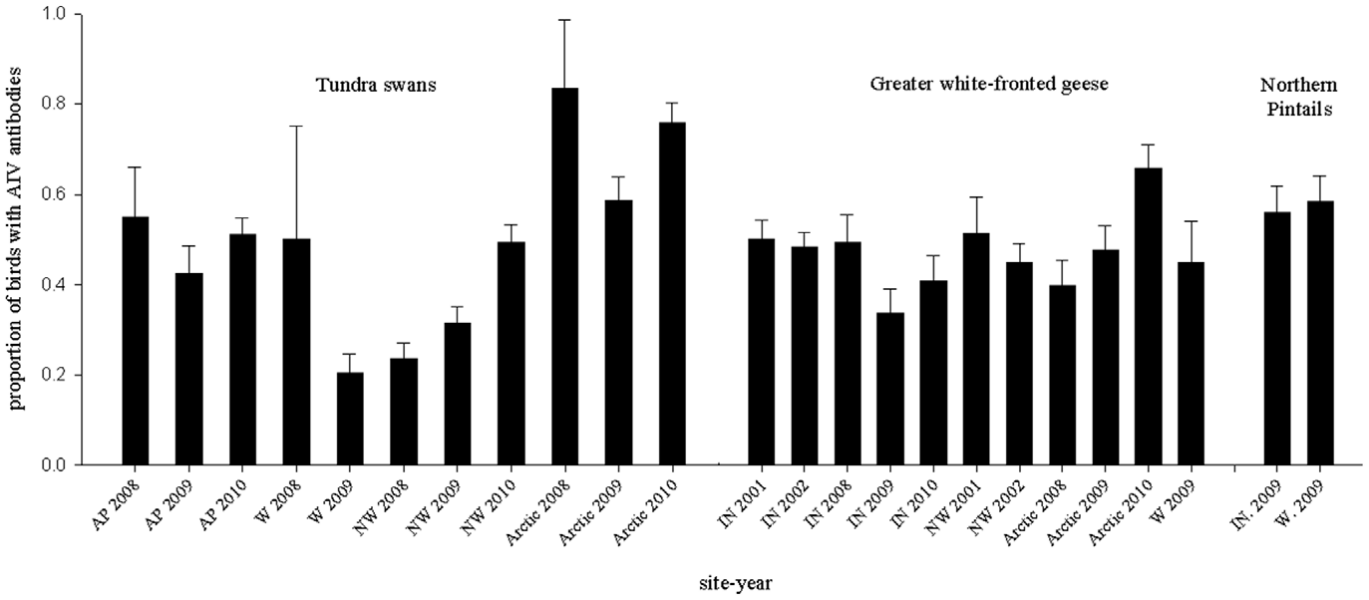
Spatio-temporal variation in avian influenza virus (AIV) seroprevalence rates (± s.e.) for tundra swans, greater white-fronted geese, and northern pintails in Alaska. Site abbreviations are as follows, AP  =  Alaska Peninsula, W  =  Western (Yukon-Kuskokwim Delta), NW = Northwestern (Kotzebue region), Arctic  =  Arctic Coastal Plain, and IN =  Interior Alaska.

AIV seroprevalence for adult waterfowl varied across species (Table 1; Fig. 2), with some evidence of differences between sexes (Table 1), but limited support for species*sex interactions (Table 1). Overall, females had higher seroprevalence rates than males (Females: 55% SE: 0.01 vs. Males: 50% SE: 0.01; Odds ratio: Females vs. Males: 1.2, 95% CI: 1.1–1.4, Table 1, model: sex + species). For the four species in which adult and sub-adult birds were sampled, our results revealed strong support for age-related differences (Table 2), with adults having 7.7 (95% CI: 5.7–10.0, Table 2, top model) times the odds of being AIV antibody positive than sub-adults. We also found support for age*species interactions (top 3 models; Table 2, Fig. 3), with SY swans and geese (seroprevalence range: 13–16%) having higher AIV antibody prevalence than HY ducks (i.e., northern pintails, 4%; Fig. 3). Our third level of examination (annual and geographic variation for tundra swans, greater white-fronted geese, and northern pintails) revealed varying patterns among species. For tundra swans (sampled 2008–2010 at AP, W, NW, and the Arctic), there was overwhelming support for variation across geographic locations (sites) and years (Table 3), with the highest seroprevalence rates occurring in the Arctic (75–83%, 2008–2010) and AP (42–55%, 2008–2010; Fig. 4). In contrast, model selection results for greater-white fronted geese (sampled 2001–2002 and 2008–2010 at IN, NW, W, and the Arctic) indicated reduced support for variation among sites and relatively little evidence of variation among years (Table 4). Similarly, results for northern pintails (sampled only in 2009 at IN and W) indicated reduced support for differences between sites (Table 5). Overall, seroprevalence rates in greater white-fronted geese and northern pintails were relatively homogenous among sites and/or years (Tables 4, 5 and Fig. 4), with the exception of a single elevated year for greater white fronted geese in the Arctic region (2010; Fig. 4).

**Table 1:** Logistic regression models of variation in avian influenza virus (AIV) seroprevalence in adult waterfowl sampled in Alaska, USA, 1998–2010 (*n* = 3,588).

Models	*k* a	AIC^b^	ΔAIC	*ω_i_* b
Species	11	4580.41	0.00	0.53
Sex + Species	12	4581.47	1.06	0.31
Sex + Species + Sex*Species	23	4582.91	2.50	0.15
Sex	1	4960.83	380.4	<0.01

a *k*  =  number of parameters in model.

b The best approximating model has the lowest Akaike's Information Criterion (AIC) value and the highest model weight (*ω_i_*), relative to others in the model set.

In this model suite, only the effects of species and sex were examined. Species include tundra swan (TUSW; *Cygnus columbianus*), cackling goose (CACG; *Branta hutchinsii*), Pacific black brant (BLBR; *B. bernicla nigricans*), greater white-fronted goose (GWFG; *Anser albifrons*), emperor goose (EMGO; *A. canagica*), northern pintail (NOPI; *Anas acuta*), Pacific common eider (COEI; *Somateria mollissima v-nigrum*), spectacled eider (SPEI; *S. fischeri*), Steller's eider (STEI; *Polysticta stelleri*), long-tailed duck (LTDU; *Clangula hyemalis*), and black scoter (BLSC; *Melanitta nigra*).

**Table 2:** Logistic regression models of variation in avian influenza virus (AIV) seroprevalence in waterfowl sampled in Alaska, USA, 1998–2010 (*n* = 3405), examining the effects of age while controlling for sex and species.

Models	*k* a	AIC^b^	ΔAIC	*ω_i_* b
Age + Sex + Species + (Age*Species)	8	3977.77	0.00	0.52
Age+ Species + (Age*Species)	7	3979.17	1.40	0.25
Age + Sex + Species + (Age*Species) + (Age*Sex)	9	3979.33	1.56	0.23
Age + Sex + Species	5	4018.27	40.50	<0.01
Age + Sex	2	4026.64	48.87	<0.01
Sex + Species	4	4278.29	300.5	<0.01

a *k*  =  number of parameters in model.

b The best approximating model has the lowest Akaike's Information Criterion (AIC) value and the highest model weight (*ω_i_*), relative to others in the model set.

Ages classes included “sub-adult”, representing hatch year (HY) birds for northern pintails and second year (SY) birds for all other species, and “adults”, representing after hatch year (AHY) birds for northern pintails and/or after second year (ASY) birds for other species. Species include tundra swan (TUSW; *Cygnus columbianus*), cackling goose (CACG; *Branta hutchinsii*), greater white-fronted goose (GWFG; *Anser albifrons*), Pacific black brant (BLBR; *B. bernicla nigricans*), and northern pintail (NOPI; *Anas acuta*).

**Table 3:** Logistic regression models of variation in avian influenza virus (AIV) seroprevalence for tundra swans sampled in Alaska, USA, 2008–2010.

Tundra swans (*n* = 1207)				
Models	*k* a	AIC^b^	ΔAIC	*ω_i_* b
Site + Year + Age + Sex	7	1474.46	0.00	0.99
Site + Age+ Sex	5	1504.28	29.82	<0.01
Year + Age + Sex	4	1523.87	49.41	<0.01
Age + Sex	7	1579.41	104.95	<0.01

a *k*  =  number of parameters in model.

b The best approximating model has the lowest Akaike's Information Criterion (AIC) value and the highest model weight (*ω_i_*), relative to others in the model set.

Models examine the effects of location (site) and/or year, while controlling for sex and age (adult vs. subadult).

**Table 4:** Logistic regression models of variation in avian influenza virus (AIV) seroprevalence for greater white-fronted geese sampled in Alaska, USA, 2008–2010.

Greater white-fronted geese (*n* = 1150)				
Models	*k* a	AIC^b^	ΔAIC	*ω_i_* b
Age + Sex	2	1540.13	0.00	0.59
Site + Age+ Sex	5	1542.61	2.48	0.17
Site + Year + Age + Sex	10	1542.91	2.78	0.15
Year + Age + Sex	7	1543.71	3.58	0.10

a *k*  =  number of parameters in model.

b The best approximating model has the lowest Akaike's Information Criterion (AIC) value and the highest model weight (*ω_i_*), relative to others in the model set.

Models examine the effects of location (site) and/or year, while controlling for sex and age (adult vs. subadult).

**Table 5:** Logistic regression models of variation in avian influenza virus (AIV) seroprevalence for northern pintails, sampled in Alaska, USA, 2009.

Northern pintails (*n* = 606)				
Models	*k* a	AIC^b^	ΔAIC	*ω_i_* b
Age + Sex	2	342.95	0.00	0.71
Site + Age+ Sex	3	344.75	1.80	0.29

Models examine the effects of location (site), while controlling for sex and age (adult vs. subadult).

a *k*  =  number of parameters in model.

b The best approximating model has the lowest Akaike's Information Criterion (AIC) value and the highest model weight (*ω_i_*), relative to others in the model set.

## Discussion

### Species and sex variation

Our data demonstrate that sampling for presence of avian influenza viruses alone may be insufficient to identify differences in AIV exposure and infection for waterfowl. For all species we sampled, seroprevalence rates far exceeded documented rates of viral shedding [16]. Several species had seroprevalences in excess of 90%, indicating that nearly the entire population had previously been infected with at least one subtype of AIV. All seroprevalence rates in excess of 60% were found in marine-dependent species. As such, our data support previous research suggesting that marine birds may have specific patterns of AIV epidemiology [24], [25]. Although our data did not permit detailed comparison of marine versus terrestrial species, evidence of differences in AIV dynamics between birds in these habitats does exist [26], and deserves further study. Additional examination of AIV dynamics, subtypes, and potential movement of virus between the marine and terrestrial ecosystems is also warranted.

While our rates of seroprevalence in geese and swans were similar to those previously published for geese and swans in Europe and Iran, many of our rates for ducks were considerably higher than those reported for ducks in France, Italy, and a range of species sampled in Argentina [19], [27]–[31]. However, differences between our study and those in Europe and South America could be due to species, geographic, or temporal differences in sampling or AIV infection patterns. Species-related variability in susceptibility to AIV's [32], as well as potential density-related transmission patterns [33]–[35], could also affect variation among species.

We found support for differences between sexes, with females having slightly higher seroprevalence rates than males; a result consistent with at least one other seroprevalence study [30]. Lower viral shedding rates were also found for females sampled in Alaska [16], perhaps suggesting a link between females' reduced susceptibility to infection and their tendency for higher seroprevalence of antibodies. We suspect gender-related differences in AIV seroprevalence rates could be the result of variation in immune response and antibody persistence rates between males and females, or could reflect sampling variation, where genders were not equally represented at particular times or locations.

### Age differences

Across many studies, prevalence of AIV infection is consistently higher in juveniles than adults [7], [16], [30], [32]. Because juveniles are generally more immunologically naïve than adults, they are also more likely to be susceptible to AIV infection. Thus, leading to higher infection and higher shedding rates [2]. Accordingly, our results demonstrated that adults have substantially greater AIV seroprevalence than sub-adults; a pattern also shown in pink-footed geese (*Anser brachyrhynchus*) and Bewick's swans (*Cygnus columbianus bewickii*; [30], [31]). Although we were not able to examine age-related patterns at finer scales of age classification (due to limitations of the data), our results did reveal that the magnitude of difference in seroprevalence rates between adults and sub-adults varied across species, with the largest difference occurring in northern pintails (53 percentage points higher in adults; Fig. 3). We suspect this species-specific variation is related to differential rates of infection, length of antibody response, and cumulative AIV exposure that also depends on species longevity; as longevity is related to repeated exposures and antibody maintenance. In our study, it is also likely that age differences were larger for northern pintails because we compared hatch year birds (with presumably more limited AIV exposure) to adults, whereas other species comparisons were between second year birds and adults. The large differences between adults and sub-adults we found suggest either long-term antibody persistence and/or very different rates of annual exposure.

### Geographic and annual variation

Among the three species with sufficient spatial replication within sampling years, only tundra swans showed strong evidence of geographic variation. We suspect two reasons for their spatial variation in seroprevalence: 1) sub-population structure and 2) migration patterns throughout the annual cycle. The tundra swan populations we sampled remain largely discrete throughout the year. Those sampled in the Arctic winter along the Atlantic coast of North America, and their higher seroprevalence could reflect higher AIV infection rates in the Atlantic Flyway. In contrast, swans from interior and western Alaska winter along the Pacific coast (primarily in California), and a proportion of the swans sampled along the Alaska Peninsula are non-migratory [36]. Further examination is needed to determine exactly where infection is occurring in this species.

We found limited support for geographic variation in greater white-fronted geese and northern pintails. Correspondingly, only moderate population segregation exists for greater white-fronted geese and little to none exists for northern pintails. White-fronted geese from the Interior and Arctic regions predominantly winter in the mid-continent of North America, whereas those from western Alaska winter in California [37], [38]. However, some overlap does exist between these populations throughout much of the annual cycle [38], and their moderate-level of population segregation appears to be mirrored in the limited geographic variation in seroprevalence we observed. Northern pintails exhibit no known consistent population structure [39], and accordingly, we found no support for geographic variation in their seroprevalence rates. As such, we contend that the spatial variation in seroprevalence we documented across species likely reflects population segregation (or lack thereof) at the time of AIV infection.

We found strong evidence of annual variation in seroprevalence only in tundra swans. Similarly, Fereidouni et al. [19] found annual variation in seroprevalence for only one of the five species (i.e., common coots; *Fulica atra*) for which they had adequate samples. We hypothesize that the annual and geographic variation we documented for tundra swans may be related to AIV circulation that occurred overwinter. Annual variation was not supported for greater white-fronted geese (Table 3), but a single site-year (Fig. 4; Arctic 2010) did indicate higher seroprevalence rates than all others. We speculate that this regional elevation in AIV activity occurred while the populations were segregated on breeding/molting areas. More frequent sampling of these populations throughout their annual cycles could help better determine spatial and temporal patterns of AIV infection.

### Antibody vs. viral prevalence

Importantly, our results, in combination with others, confirm that the number of birds that have experienced previous AIV infections is much higher than that indicated by current prevalence rates (i.e., those based on rRT-PCR or virus isolation from swab samples alone [19]). Further, our results of high seroprevalences, but low infection rates, suggest that most infections for the birds in our sample occurred at other times of the year. However, further analysis and surveillance are needed to adequately address this hypothesis. Although waterfowl have been associated with numerous HPAIV outbreaks, mortality has typically been limited in wild birds [40]–[43]. This could be due to lack of exposure. However, laboratory studies [9], [13] suggest individual immunological history is also an important factor, among others, that may modify rates of AIV-related infection and mortality. Given some LPAIV antibodies can provide cross-protection to HPAIV H5N1, our results suggest that a relatively large proportion of waterfowl populations in Alaska could have immunity or develop only limited HPAIV infections (i.e., they could become disease carriers without debilitating clinical signs), while immunologicaly naïve birds might be expected to serve as sentinels [8], [9], [13], [18], [44], [45]. Under this scenario, we question some inferences drawn from experimentally infected, immunologically naïve birds in captivity for predicting pathogenicity of HPAIV H5N1 in wild birds [3], [4], [46]. Results from captive studies using immunologically naïve birds (in which most birds died), have suggested that wild birds may not live long enough to transmit the virus, and thus, would not be a viable mechanism for dispersal. However, our results, in combination with others [30] indicate that wild, adult birds have relatively high rates of natural AIV exposure, with the related possibility of immunity, potentially allowing at least some birds to survive HPAIV infection and play a role in further transmission [13].

### Implications for future surveillance

We believe our seroprevalence findings offer another point of insight into the myriad of factors influencing differential survival associated with HPAI outbreaks in the wild and have important implications for future monitoring and surveillance for HPAIV. Given seroprevalence is consistently higher than virus shedding rates, we believe monitoring virus shedding alone substantially underestimates the true number of birds experiencing AIV infection, and thus, may not be an adequate stand-alone tool for prioritizing species for surveillance sampling. Further, we believe that without seroprevalence information, species with low rates of shedding could be prematurely dismissed in surveillance plans. Species with low rates of shedding, but high seroprevalence, may be potential HPAIV carriers, and hence deserve further consideration. Seroprevalence information could also be used to exclude some species from future live-bird surveillance. For example, species with low rates of AIV infection *and* low prevalence of antibodies could more safely be considered unlikely reservoir hosts, and thus, excluded from further monitoring. However, the AIV-naïve species may be good sentinels during HPAIV mortality events because they have limited potential for cross-immunity. Overall, our results, in concert with those from other AIV antibody prevalence studies [19], [29], [47], suggest that surveillance for virus shedding *alone* may provide an incomplete picture of transmission potential relative to surveys which also include antibodies.

Seroprevalence is a complex measure that involves cumulative infection, cross-immunity, and antibody lifetime [30]. While it clearly indicates where infection has occurred and can provide a good indication of potential host species, it also comes with caveats that can limit inference [30], [48]. Without clear knowledge of transmission or antibody persistence patterns, identification of reservoir hosts using seroprevalence alone can be limited [48]. Furthermore, interpretation of seroprevalence in relation to HPAIV surveillance can be difficult. For example, birds without previous exposure to AIV (or short-term antibody persistence) may be more susceptible to HPAIV (i.e., given lack of cross-protective antibodies), and thus, become important indictors of an HPAIV outbreak. However, higher HPAIV mortality may also render them inefficient in long-range disease transmission. In contrast, a portion of birds with LPAIV antibodies may have a higher likelihood of surviving HPAIV infection, and therefore, more opportunity for transmission beyond localized hotspots. While these sero-positive birds might have reduced durations and rates of viral shedding (shortening the window for detecting HPAIV [45]), their HPAIV infections might be detectable with subtype-specific AIV serology.

The balance between allocating surveillance resources to detection of virus versus antibodies, as well as appropriate ways to combine the two, will require careful consideration. However, we believe populations with high seroprevalence rates (even those with low shedding rates), should be included in surveillance plans, as these species have demonstrated a high potential for contracting AIV infections. Serologic testing for antibody presence, coupled with serotype specific antibody testing, could 1) provide information on prevalence and AIV subtypes of previous exposure, 2) help determine antibody longevity and cross-protection (e.g., [10]), and 3) enhance overall information on rates of infection and disease dynamics. Therefore, we recommend future surveillance plans include seroprevalence information in developing measures of AIV infection to assess risk for a species. We also suggest that key species with high seroprevalence rates are sampled throughout the annual cycle, to identify periods and places of transmission and illness (e.g., [18], [30]). Finally, our understanding of factors which influence infection rates would be greatly improved by examining both serologic and infection data relative to aggregation patterns (bird density), wintering areas, susceptibilities, average life-spans, terrestrial versus marine habits, and environmental reservoirs.

## References

[1] Keawcharoen J, van Riel D, van Amerongen G, Bestebroer T, Beyer WE, et al (2008) Wild ducks as long-distance vectors of highly pathogenic avian influenza virus (H5N1). Emerging Infectious Diseases 14: 600–607. 18394278 10.3201/eid1404.071016 PMC2570914

[2] Webster RG, Bean WJ, Gorman OT, Chambers TM, Kawaoka Y (1992) Evolution and ecology of influenza A viruses. Microbiological Reviews 56: 152–179. 1579108 10.1128/mr.56.1.152-179.1992 PMC372859

[3] Brown JD, Stallknecht DE, Beck JR, Suarez DL, Swayne DE (2006) Susceptibility of North American ducks and gulls to H5N1 highly pathogenic avian influenza viruses. Emerging Infectious Diseases 12: 1663–1670. 17283615 10.3201/eid1211.060652 PMC3372354

[4] Brown JD, Stallknecht DE, Swayne DE (2008) Experimental infection of swans and geese with highly pathogenic avian influenza virus (H5N1) of Asian lineage. Emerging Infectious Diseases 14: 136–142. 18258093 10.3201/eid1401.070740 PMC2600149

[5] Globig A, Staubach C, Beer M, Köppen U, Fielder W, et al (2009) Epidemiological and ornithological aspects of outbreaks of highly pathogenic avian influenza virus H5N1 of Asian lineage in wild birds in Germany, 2006–2007. Transboundary and Emerging Diseases 56: 57–72. 19267878 10.1111/j.1865-1682.2008.01061.x

[6] Kou Z, Li Y, Yin Z, Guo S, Wang M, et al (2009) The survey of H5N1 flu virus in wild birds in 14 provinces of China from 2004 to 2007. PLoS ONE 4: e6926. 19742325 10.1371/journal.pone.0006926 PMC2735031

[7] Pybus OG, Perrins CM, Choudhury B, Manvell RJ, Nunez A, et al (2012) The ecology and age structure of a highly pathogenic avian influenza virus outbreak in wild mute swans. Parasitology 139: 1914–1923. 22339986 10.1017/S0031182012000261

[8] Pasick J, Berhane Y, Embury-Hyatt C, Copps J, Kehler H, et al (2007) Susceptibility of Canada geese (*Branta canadensis*) to highly pathogenic avian influenza virus (H5N1). Emerging Infectious Diseases 13: 1821–1827. 18258030 10.3201/eid1312.070502 PMC2876756

[9] Kalthoff D, Breithaupt A, Teifke JP, Globig A, Harder T, et al (2008) Highly pathogenic avian influenza virus (H5N1) in experimentally infected adult mute swans. Emerging Infectious Diseases 14: 1267–1270. 18680652 10.3201/eid1408.080078 PMC2600380

[10] Fereidouni SR, Starick E, Beer M, Wilking H, Kalthoff D, et al (2009) Highly pathogenic avian influenza virus infection of mallards with homo- and heterosubtypic immunity induced by low pathogenic avian influenza viruses. PLoS ONE 4: e6706. 19693268 10.1371/journal.pone.0006706 PMC2724736

[11] Fereidouni SR, Grund C, Hauslaigner R, Lange E, Wilking H, et al (2010) Dynamics of specific antibody responses induced in mallards after infection by or immunization with low pathogenicity avian influenza viruses. Avian Diseases 54: 79–85. 20408403 10.1637/9005-073109-Reg.1

[12] Berhane Y, Leith M, Embury-Hyatt C, Neufeld J, Babiuk S, et al (2010) Studying possible cross-protection of Canada geese preexposed to North American low pathogenicity avian influenza virus strains (H3N8, H4N6, H5N2) against an H5N1 highly pathogenic avian influenza challenge. Avian Diseases 54: 548–554. 20521692 10.1637/8841-040309-Reg.1

[13] Costa TP, Brown JD, Howerth EW, Stallknecht DE, Swayne DE (2011) Homo- and heterosubtypic low pathogenic avian influenza exposure on H5N1 highly pathogenic avian influenza virus infection in wood ducks (*Aix sponsa*). PLoS ONE 6: e15987. 21253608 10.1371/journal.pone.0015987 PMC3017094

[14] Jourdain E, Gunnarsson G, Wahlgren J, Latorre-Margalef N, Bröjer C, et al (2010) Influenza virus in a natural host, the mallard: experimental infection data. PLoS ONE 5: e8935. 20126617 10.1371/journal.pone.0008935 PMC2812492

[15] Winker K, McCracken KG, Gibson DD, Pruett CL, Meier R, et al (2007) Movements of birds and avian influenza from Asia into Alaska. Emerging Infectious Diseases 13: 547–552. 17553268 10.3201/eid1304.061072 PMC2725966

[16] Ip HS, Flint PL, Franson JC, Dusek RJ, Derksen DV, et al (2008) Prevalence of Influenza A viruses in wild migratory birds in Alaska: Patterns of variation in detection at a crossroads of intercontinental flyways. Virology Journal 5: 71. 18533040 10.1186/1743-422X-5-71 PMC2435106

[17] Hénaux V, Samuel MD (2011) Avian influenza shedding patterns in waterfowl: implications for surveillance, environmental transmission, and disease spread. Journal of Wildlife Diseases 47: 566–578. 21719821 10.7589/0090-3558-47.3.566

[18] Hénaux V, J.Parmely, Soos C, Samuel MD (2013) Estimating tansmission of avian influenza in wild birds from incomplete epizootic data: implications for surveillance and disease spread. Journal of Applied Ecology 50: 223–231.

[19] Fereidouni SR, Werner O, Starick E, Beer M, Harder TC, et al (2010) Avian influenza virus monitoring in wintering waterbirds in Iran, 2003–2007. Virology Journal 7: 43. 20167132 10.1186/1743-422X-7-43 PMC2837633

[20] Brown JD, Stallknecht DE, Berghaus RD, Luttrell MP, Velek K, et al (2008) Evaluation of a commercial blocking enzyme-linked immunosorbent assay to detect avian influenza virus antibodies in multiple experimentally infected avian species. Clinical and Vaccine Immunology 16: 824–829. 10.1128/CVI.00084-09 PMC2691043 19386796

[21] Spackman E, Pantin-Jackwood MJ, Swayne DE, Suarez DL (2009) An evaluation of avian influenza diagnostic methods with domestic duck specimens. Avian Diseases 53: 276–280. 19630236 10.1637/8520-111708-Reg.1

[22] USFWS/USGS (2009–2011) Sampling for highly pathogenic Asian H5N1 avian influenza in migratory birds in Alaska: results of 2008–2010 field seasons. Progress Reports. U.S. Fish and Wildlife Service (Region 7, Alaska) U.S. Geological Survey, Alaska Science Center, Anchorage, Alaska and U.S. Geological Survey, National Wildlife Health Center, Madison, Wisconsin. http://alaska.usgs.gov/science/biology/avian_influenza/pubs.php.

[23] Burnham KP, Anderson DR (2002) Model selection and multimodel inference: a practical information theoretic approach 2nd edition. New York, NY: Springer-Verlag.

[24] Sazonov AA, Lvov DK, Webster RG, Sokolova TV, Braude NA, et al (1977) Isolation of an influenza virus, similar to A/Port Chalmers/1/73/(H3N2) from a common murre at Sakhalin Island in U.S.S.R. (strain A/CommonMurre/Sakhalin1/74). Archives of Virology 53: 1–7. 851395 10.1007/BF01314842

[25] Granter A, Wille M, Whitney H, Robertson GJ, Ojkic D, et al (2010) The genomic sequence of an H11N2 avian influenza virus from a thick-billed murre (*Uria lomvia*) shows marine-specific and regional patterns of relationships to other viruses. Virus Genes 41: 224–230. 20582460 10.1007/s11262-010-0504-5

[26] Ramey AM, Pearce JM, Reeves AB, Franson JC, Petersen MR, et al (2011) Evidence for limited exchange of avian influenza viruses between seaducks and dabbling ducks at Alaska Peninsula coastal lagoons. Archives of Virology 156: 1813–1821. 21766196 10.1007/s00705-011-1059-z

[27] De Marco MA, Foni GE, Campitelli L, Raffini E, Trani LD, et al (2003) Circulation of influenza viruses in wild waterfowl wintering in Italy during the 1993–99 period: evidence of virus shedding and seroconversion in wild ducks. Avian Diseases 47: 861–866. 14575078 10.1637/0005-2086-47.s3.861

[28] Niqueux E, Guionie O, Schmitz A, Hars J, Jestin V (2010) Presence of serum antibodies to influenza A subtypes H5 and N1 in swans and ibises in French wetlands, irrespective of highly pathogenic H5N1 natural infection. Avian Diseases 54: 502–508. 20521685 10.1637/8804-040109-ResNote.1

[29] Brown JD, Luttrell MP, Uhart MM, Ferreyra HDV, Romano MM, et al (2010) Antibodies to type A influenza virus in wild waterbirds from Argentina. Journal of Wildlife Diseases 46: 1040–1045. 20688720 10.7589/0090-3558-46.3.1040 PMC11337144

[30] Hoye BJ, Munster VJ, Nishiura H, Fouchier RAM, Madsen J, et al (2010) Reconstructing an annual cycle of interaction: natural infection and antibody dynamics to avian influenza along a migratory flyway. Oikos 120: 001–008.

[31] Hoye BJ, Fouchier RAM, Klaassen M (2011) Host behavior and physiology underpin individual variation in avian influenza virus infection in migratory Bewick's swans. Proceedings of the Royal Society B 279: 529–534. 21733894 10.1098/rspb.2011.0958 PMC3234555

[32] Stallknecht DE, Shane SM (1988) Host range of avian influenza virus in free-living birds. Veterinary Research Communications 12: 125–141. 3055662 10.1007/BF00362792

[33] McCallum H, Barlow N, Hone J (2001) How should pathogen transmission be modelled? Trends in Ecology and Evolution 16: 295–300. 11369107 10.1016/s0169-5347(01)02144-9

[34] Krauss S, Stallknecht DE, Negovetich NJ, Niles LJ, Webby RJ, et al (2010) Coincident ruddy turnstone migration and horseshoe crab spawning creates an ecological ‘hot spot’ for influenza viruses. Proceedings of the Royal Society B 277: 3373–3379. 20630885 10.1098/rspb.2010.1090 PMC2982236

[35] Gaidet N, Caron A, Cappelle J, Cumming GS, Balanca G, et al (2011) Understanding the ecological drivers of avian influenza virus infection in wildfowl: a continental-scale study across Africa. Proceedings of the Royal Society B 279: 1131–1141. 21920984 10.1098/rspb.2011.1417 PMC3267134

[36] Dau CP, Sarvis JE (2002) Tundra swans of the lower Alaska Peninsula: differences in migratory behavior and productivity. Waterbirds 25: 241–249.

[37] Ely CR, Dzubin AX (1994) Greater white-fronted goose (*Anser albifrons*) Poole A, Gill F, editors: The Birds of North America. Academy of Natural Sciences, Philadelphia, PA and American Ornithologists' Union, Washington, D.C., USA.

[38] Ely CR, Takekawa JY (1996) Geographic variation in migratory behavior of greater white-fronted geese (*Anser albifrons*). Auk 113: 889–901.

[39] Flint PL, Ozaki K, Pearce JM, Guzzetti B, Higuchi H, et al (2009) Breeding season sympatry facilitates genetic exchange among allopatric wintering populations of northern pintails in Japan and California. Condor 111: 591–598.

[40] Nagy A, Machova J, Hornickova J, Tomci M, Nagl I, et al (2007) Highly pathogenic avian influenza virus subtype H5N1 in mute swans in Czech Republic. Veterinary Microbiology 120: 9–16. 17113249 10.1016/j.vetmic.2006.10.004

[41] Uchida Y, Mase M, Yoneda K, Kimura A, Obara T, et al (2008) Highly pathogenic avian influenza virus (H5N1) isolated from whooper swans, Japan. Emerging Infectious Diseases 14: 1427–1429. 18760011 10.3201/eid1409.080655 PMC2603097

[42] Hars J, Ruette S, Benmergui M, Fouque C, Fournier JY, et al (2008) The epidemiology of the highly pathogenic H5N1 avian influenza in mute swan (*Cygnus olor*) and other *Anatidae* in the Dombes region (France), 2006. Journal of Wildlife Diseases 44: 811–823. 18957637 10.7589/0090-3558-44.4.811

[43] Śmietanka K, Minta Z, Domańska-Blicharz K, Tomczyk G, Wijaszka T (2008) Avian influenza H5N1 outbreak in a flock of mute swans in the city of Toruń, Poland, in 2006. Bulletin of the Veterinary Institute Pulawy 52: 491–495.

[44] Khalenkov A, Perk S, Panshin A, Goldender N, Webster RG (2009) Modulation of severity of highly pathogenic H5N1 influenza in chickens previously inoculated with Israeli H9N2 influenza viruses. Virology 383: 32–38. 18992907 10.1016/j.virol.2008.09.026 PMC2632966

[45] Hénaux V, Samuel MD, Bunck CM (2010) Model-based evaluation of highly and low pathogenic avian influenza dynamics in wild birds. PLoS ONE 5: e10997. 20585637 10.1371/journal.pone.0010997 PMC2890401

[46] Brown JD, Stallknecht DE, S.Valeika, Swayne DE (2007) Susceptibility of wood ducks to H5N1 highly pathogenic avian influenza virus. Journal of Wildlife Diseases 43: 660–667. 17984261 10.7589/0090-3558-43.4.660

[47] Brown JD, Luttrell MP, Berghaus RD, Kistler W, Keeler SP, et al (2010) Prevalence of antibodies to type A influenza virus in wild avian species using two serologic assays. Journal of Wildlife Diseases 46: 896–911. 20688695 10.7589/0090-3558-46.3.896 PMC11345711

[48] Haydon D, Cleaveland S, Taylor L, Laurenson M (2002) Identifying reservoirs of infection: a conceptual and practical challenge. Emerging Infectious Diseases 8: 1468–1473. 12498665 10.3201/eid0812.010317 PMC2738515

